# Influences on trust in the use of AI-based triage—an interview study with primary healthcare professionals and patients in Sweden

**DOI:** 10.3389/fdgth.2025.1565080

**Published:** 2025-05-20

**Authors:** Emilie Steerling, Petra Svedberg, Per Nilsen, Elin Siira, Jens Nygren

**Affiliations:** ^1^ School of Health and Welfare, Halmstad University, Halmstad, Sweden; ^2^ Department of Health, Medicine and Caring Sciences, Linköping University, Linköping, Sweden

**Keywords:** trust, artificial intelligence, AI-based triage, primary care, interview study

## Abstract

**Introduction:**

Artificial intelligence (AI) has the potential to improve the quality and efficiency of medical triage in primary care. However, there are many uncertainties related to its use. Trust in these systems is important for successful integration and advancement into healthcare, yet this remains an understudied issue. Understanding the influences on trust in the actual use of AI is necessary for developing effective implementation strategies.

**Objective:**

This study aimed to explore the influences on trust of healthcare professionals and patients in the use of AI-based triage in primary care in Sweden.

**Methods:**

We applied qualitative study design using an inductive approach based on semi-structured interviews with 14 healthcare professionals and 12 patients in two regions in Sweden. The participants had experience of using AI-based triage in primary care. The interviews were transcribed verbatim and analyzed with reflexive thematic analysis to explore the influences on trust.

**Results:**

Healthcare professionals and patients experienced three types of influences on their trust in the use of AI-based triage in primary care: (1) provision of accurate patient information, (2) alignment with clinical expertise, and (3) supervision of patients’ health and safety. Their experiences across these themes varied only in terms of the influence of experience-based knowledge. Both healthcare professionals and patients emphasized the importance of constructive dialogue, along with clear instructions for the use and storage of information.

**Conclusions:**

The results demonstrate that building trust in AI requires improved interaction to ensure that the system is adapted to the users' competencies and level of expertise. The generalizability of these insights is limited to AI-based triage in primary care in Sweden. Future research should explore trust in AI across different healthcare settings to inform policy, as well as to ensure safe use and design of AI applications.

## 1 Introduction

The use of artificial intelligence (AI) to support or automate clinical decision-making is radically changing healthcare (1). The demands on healthcare is rising (2–4) due to demographic shifts, such as an ageing population, an increasing prevalence of chronic diseases, and declining healthcare workforce (5, 6). These challenges on working conditions have resulted in concerns related to the quality of care and the risk of compromising patients' safety (7–9). Medical triage has traditionally been part of nurses' work in primary care, where the nurses direct the patients according to their symptoms and level of urgency to ensure appropriate care, in the right place, and at the right time (10). Against this background, AI-based triage systems have the potential to automate medical history-taking, suggest diagnoses, and direct patients to appropriate care pathways, thereby reducing the workload of healthcare professionals while improving efficiency and quality of care (11–13).

However, several barriers hinder the implementation of AI-based triage (11, 14–16) with the predominant concerns focusing on their accuracy and performance (12–14, 17–20). Furthermore, research has investigated healthcare professionals' and patients' perceptions and attitudes toward these systems (21, 22), as well as their impact on healthcare professionals’ judgment. Some findings have suggested that AI can augment healthcare professionals' decision-making with improved performance (23), while others have highlighted the risk of confirmation bias and the importance of human control (24, 25). Notably, several researchers (21, 24) have emphasized the importance of trust in healthcare settings, given the uncertainties in the AI use, and the considerable risks associated with erroneous decision-making.

Although the importance of trust in the context of AI use in healthcare is widely acknowledged, interdisciplinary research, from psychology, sociology and computer science (26–28), has not produced a unified understanding of its meaning and influences. Emphasis is often placed on either trust in technology, interpersonal trust, or trust in institutions. The different definitions to trust depend on its complexity and multidimensional nature (26). Reliance and dependence are frequently highlighted as key components of trust (29), closely tied to an individual's perceived sense of control (30). Trust is also commonly viewed as an attituded grounded in vulnerability and positive expectations (31), directed toward those who are believed to have compelling reasons to act in the trustor's best interest (32). One common definition of trust in the interaction between humans and AI is: “the willingness of a party to be vulnerable to the actions of another party based on the expectations that the other will perform a particular action important to the trustor, irrespectively of the ability to monitor or control that other party” [(33), p. 712].

Transparency and explainability of AI's output are widely regarded essential for fostering trust in human-AI interactions (34). The inability to critically evaluate AI-generated outputs can result in over-reliance, while also raising significant ethical and legal concerns (35). According to the human-computer trust (HCT) model (36), trust is not only shaped by the user's cognitive evaluation but also the user's emotional responses. Cognition-based trust is comprised by perceived understandability, perceived technical competence and perceived reliability, whereas affect based trust comprises faith and personal attachment. In situations where decision-makers have limited knowledge or information, these emotional responses become more important.

Previous empirical research on trust in AI have often applied and modified technology adoption models (37–41), such as the Technology Acceptance Model (TAM) (42) and the Unified Theory of Acceptance and Use of Technology (UTAUT) (43). However, to adequately address the numerous challenges, uncertainties and perceived risks in the use of AI, a more holistic approach is needed (44) where trust is understood in relation to specific sociotechnical contexts (45). Unlike other digital technologies, AI has the capacity to provide insights from complex datasets with a level of performance that can, at times, surpass human capabilities (46). This unique characteristic has prompted discussions regarding AI's epistemic status (47–51). Hence, human–AI interaction needs to be understood within the broader social system of healthcare where the different sets of knowledge and value systems shape practices and interpretations (52).

This study considered AI-based triage in primary care from a sociotechnical perspective with a focus on the specific context of primary care triage. The aim was to gain a deeper understanding of trust and thereby to facilitate successful integration of AI in triage processes. Most previous research has concentrated on the hypothetical use of AI systems designed for medical history taking and triage, while few studies have explored real-world experiences of healthcare professionals and patients with these systems in practice (14, 53), particularly from the patients' perspectives (21). Patients and healthcare professionals face distinct vulnerabilities in the use of AI-based triage, as patients require appropriate care while healthcare professionals have the responsibility of its delivery (54). To our knowledge, there are no previous studies exploring the trust in AI-based triage in primary care of both healthcare professionals and patients based on their actual experiences of using the application. Understanding the influences on trust in AI-based triage provides a basis for developing strategies for effective implementation and use of AI for triage purposes.

The AI triage system in this study is an application that comprises software designed to automate medical history-taking and triage in primary care, as well as to support decision-making and patient management. A detailed description of the application is available in a previous publication (15). The system is based on a Bayesian network that is constantly updated and improved by a medical team based on healthcare professionals' feedback. The AI-based triage process starts with the patients describing their symptoms in an automated chat, where a series of questions follows based on their previous answers. The healthcare professionals receive a report generated by the AI application when this process has been completed. The information from this report presents the patients' symptoms, urgency, and potential diagnoses. The AI application only functions as a decision support and healthcare professionals are responsible for the delivery of appropriate care.

The aim of this study was thus to explore the influences on trust of healthcare professionals and patients in the use of AI-based triage in primary care in Sweden.

## 2 Methodology

### 2.1 Design

The aim was to reach a deep understanding of participants' experiences of using AI-based triage in primary care. Experience in this context was understood as an expression of the cognitive processes that arise when using the AI-based triage, involving intellectual, emotional, moral, and social aspects (55). Furthermore, reflexive thematic analysis (56) was employed to address the study aim, as its flexible and interpretative nature enables in-depth and exploration of the complexity of human behavior and social phenomena. Data were collected through semi-structured interviews, a method that allowed for both flexibility in probing relevant topics and sufficient structure to ensure consistency across interviews (57). The Reflexive Thematic Analysis Reporting Guidelines (RTARG) were used to report the methods (58, 59).

### 2.2 Setting

The Swedish healthcare system is publicly funded through taxes and managed by 21 regional councils with the goal to provide good health and care on equal terms to the entire population. Healthcare professionals in primary care offer medical assessment and treatment, nursing care, preventive work, and rehabilitation (60). “Good quality, local health care” (61) is a reform to strengthen the role of primary care with an emphasis on digitalization and person-centered care, to ensure accessible care based on the patients' individual needs, conditions, and resources. This reform is in line with AI-based triage in primary care with the aim of improving accessibility to care by reducing travel and waiting time, as well as saving time for healthcare professionals and improving engagements with patients. Digital consultations have been used within primary care since 2016 in the context of the Patient Act's deregulation of care provision (62). The AI-based triage application had been implemented in four regions within primary care in Sweden at the time of the data collection. This study was conducted in two regions located in southern Sweden.

### 2.3 Participant recruitment

Interviews were conducted with a total of 26 participants from two regions. The inclusion criteria were as follows: (1) participants must be either a primary care professional or a patient aged 18 or older within one of the selected regions, (2) participants must have practical experience with the implementation or use of the AI application, and (3) proficiency in the Swedish language.

Among the 26 participants, 14 were primary care professionals, and 12 were patients. The healthcare professionals consisted of two psychologists, eight doctors, three registered nurses, and one counselor, with nine women and five men. Of the healthcare professionals, nine were from region 1 and five from region 2. Their experience with the AI application ranged from 3 weeks to 3 years.

The 12 patients had various health conditions, including sleep problems, coughs, insect bites, urinary tract infections, anxiety, arm pain, and rashes. Eight of the patients were using the AI application for the first time to seek care in primary care, while four had prior experience with the application. The patients consisted of seven women and five men, with nine patients from region 1 and three from region 2. See Table 1 for participant characteristics.

**Table 1:** Participant characteristics.

Characteristic	Patients	Healthcare professionals	Total
Sex			
Men	5	5	10
Women	7	9	16
Age (years)			
Range	22–66	28–64	–
Mean	39.35	45	–
Educational level			
Secondary	3	0	3
Tertiary	9	14	23
Occupational status			
Employed	10	14	24
Retired/unemployed	2	0	2
Profession			
Nurse	–	3	3
Physician	–	8	8
Psychologist	–	2	2
Other	–	1	1
Previous experience of using the AI application			
Yes	4	14	–
No	8	–	–
Duration of using the AI application at primary care unit			
3 years	–	14	–
11–18 months 2	–	0	–
3 months	–	0	–
Region			
Region 1	9	9	18
Region 2	3	5	8
**Total**	**12**	**14**	**26**

The recruitment process was conducted in two distinct ways for primary care professionals and patients. To recruit primary care professionals, we contacted one primary care manager from each region and asked if they would be willing to allow professionals with experience using the AI-based triage application to participate in the study. The managers were asked to provide the researchers with contact details of employees that represented a range of genders, occupational backgrounds, and experience levels with the AI application. A total of 15 primary care professionals were contacted and provided with written information about the study. One individual declined to participate, leaving a total of 14 participants.

Patients were recruited through the AI application. After their case was closed, they received a message with brief information about the study and were asked to provide their contact details if they were interested in participating. As a result, 71 individuals submitted their contact information and were sent more detailed information about the study via phone or email. We sought to recruit 15 patients. 58 patients either declined or did not respond after receiving more information. 13 participants expressed a willingness to participate, but one was excluded because they could not recall using the AI application.

All the participants were informed both orally and in writing about the purpose and procedures of the study and had also the opportunity to ask questions.

### 2.4 Data collection

Data was collected using semi-structured interviews via video communication between February and April 2023. The interview guide included open-ended questions to allow exploration of unforeseen topics. The content of the guide was the same for both patients and healthcare professionals, but the wording of the questions was adapted to suit each group. The questions for healthcare professionals were structured around (i) the professional responsibilities; (ii) AI's impact on the clinical decision-making; (iii) on the care meeting, (iv) and AI's role in this process. A pilot interview was conducted with a healthcare professional with experience of using the AI-based triage application to make sure the questions reflected the aim of the study and were not included in the analysis. The interviews with healthcare professionals lasted an average of 41 (31–57) minutes. The questions for patients focused on (i) the reason for seeking care; (ii) the experience of using the AI-based triage application; (iii) how the questions were perceived; (iv) the impact on the care meeting; (v) patient safety considerations. A pilot interview was conducted with a patient with experience of using the AI-based triage application to make sure the questions reflected the topic and were not included in the study. The interviews with patients lasted an average of 47 (41–58) minutes.

All the interviews were conducted by two researchers, ESi (PhD, postdoctoral researcher) and DT (PhD, senior associate lecturer) who both have training and experience in qualitative methods. They had no pre-existing relationship with the participants. All the interviews were audio-recorded and transcribed verbatim.

### 2.5 Data analysis

In order to gain a comprehensive understanding of the data and to capture the complexity of influences on trust in AI-based triage in primary care, the data were analyzed following the six phases of reflexive thematic analysis as described by Braun and Clarke (56): (1) data familiarization; (2) initial code generation; (3) generating themes; (4) theme review; (5) theme defining and naming; (6) and report production. We utilized an inductive approach where coding and theme generation were informed by the healthcare professionals and patients' subjective experiences.

The first step involved reading the interviews several times, as well as taking notes. To achieve greater contextual understanding of the data, the audio recordings were listened to, and key notes were taken. One author (ES) reflected and generated lists of codes from words and phrases, which captured the influences on trust in the use of AI-based triage in primary care of healthcare professionals and patients. Influences were defined as internal or external factors that affect, shape, or change the behavior, development, or outcome of something. The coding stayed close to the participants' understanding of their experiences. The author (ES) then reflected on the relationship between the codes and grouped them into preliminary subthemes. The co-authors (ES, JN, PN, PS, ESi) provided their interpretations and the relationship between the codes were discussed. The codes developed throughout the coding process, as the authors' understanding of the subject developed. Thereafter, the subthemes were analyzed, and three themes were generated.

A thematic map was used to illustrate the relationships between codes and themes. All authors (ES, JN, PN, PS, ESi) discussed the data analysis in regular meetings throughout the process to enhance quality and validity. Coding quality in this approach to thematic analysis did not stem from consensus, but the depth of engagement with the data and interpretations, which made saturation difficult to align (56). The authors' different backgrounds provided a multidisciplinary perspective, comprising nursing (PS), implementation science (PN), sociology (ESi), and health sciences (ES, JN), where the different perspectives and experiences gave richer interpretations of meanings. The quotations in the study were translated from Swedish to English. There were some minor changes to the quotations to make them easier to understand, but without changing the meaning of the statements. No qualitative data analysis software was used.

### 2.6 Ethical consideration

The study was conducted in accordance with the World Medical Association Declaration of Helsinki ethical principles for medical research involving human subjects (63) and received approval by the Swedish Ethical Review Authority (no. 2022-04787-01). All the participants gave their informed consent to take part in the study and were free to withdraw at any time without needing to provide a reason.

## 3 Results

Three types of influences on trust in the use of AI-based triage were generated from the analysis of the interviews with healthcare professionals and patients. These themes were labeled: provision of accurate patient information, alignment with clinical expertise, and supervision of patients' health and safety (Table 2). The themes are presented below with representative quotes for each theme, showing differences and similarities between healthcare professionals and patients. Healthcare professionals and patients are referred to as HCP and P, respectively, along with the individual number assigned to each participant, in connection with their quotes (e.g., HCP1 and P1).

**Table 2:** Influences on trust in the use of AI-based triage.

Themes	Subthemes	Characteristics
Provision of accurate patient information	Patient's capability to provide accurate information	Patient's necessary qualities for providing accurate information
	Patient's willingness to provide accurate information	Patient's quality or state of being happy about providing accurate information
Alignment with clinical expertise	Standardized reasoning	AI-based triage directs the user's attention by following a medical logic, starting from high priority to low
		Healthcare professionals’ knowledge is based on their subjective experiences
	Experience-based knowledge (only healthcare professionals)	
Supervision of patients’ health and safety	Professionalism as protection against inaccurate information	Healthcare professionals in the healthcare system protect patients from injury or harm in the use of AI-based triage as they have the expected competence and skills
	Guidance in the use of the information	Help and advice about how to use the information

### 3.1 Provision of accurate patient information

Trust in the AI application is influenced by the participants' experiences concerning the patients' capability and willingness to provide accurate information when describing their symptoms in the automated chat. This was crucial as the report from the AI application was restricted to the information provided by the patients in the chat.

#### 3.1.1 Patients' capability to provide accurate information

Both healthcare professionals and patients experienced that trust in AI-based triage is influenced by the patients' own understanding of their symptoms and well-being, and that they do not always have sufficient knowledge to know what information healthcare professionals need in order to make an accurate assessment. As one healthcare professional explained, some patients may ignore answering the AI-based triage application's questions properly if they experience them as irrelevant. Healthcare professionals and patients both agreed that some health conditions were more suitable for AI-based triage as they were easier to communicate.

“…it's easier to explain facts such as the stomach hurting compared to the mind hurting.” [HCP 3]

“my symptoms were very simple, and I had clear answers to the questions (…) if I would have had stomach pain and no appetite it probably wouldn't have been as easy to answer”. [P 3]

Both healthcare professionals and patients experienced that the patients' language skills and technical skills influenced how they provided information when seeking care. Some patients explained that their previous experience from using similar AI applications in other contexts facilitated the use of this application. From the patients' perspective, the interaction with the AI application focused on keywords, which for experienced patients facilitated the provision of information while for those with less skills, complicated the process.

“And I know how the tool works so I've appreciated it and made maximum use of it. I've really been able to write so that the doctor is ready. Then it works very effectively, managing many things because I knew the system, but patients don”t know it.” [HCP 1]

“I knew, so I could use keywords, which it probably is based upon (…) this probably made it easier for me”. [P 3]

Another worry referred to by both healthcare professionals and patients concerned the number of questions that patients had to answer in order to reach a healthcare professional, since many patients often lack persistence.

#### 3.1.2 Patients' willingness to provide accurate information

Both healthcare professionals and patients experienced the risk of patients being dishonest in their provision of information. Some healthcare professionals explained that they cannot blindly trust the AI application, because it is not based on facts and patients may ignore answering questions. From the patients' perspective, the large number of questions made them fear that they would never consult a healthcare professional. Hence, in order to get higher priority, they exaggerated the symptoms. This problem was echoed by healthcare professionals.

“The patient must understand that they must write in a certain way in order to see a doctor. It must never be perceived as a struggle.” [HCP 1]

“It's a question of answering correctly otherwise they'll refer you to a website where there is sleep advice. It was such a feeling I had which was based on previous experiences (…) it's easy to think that you have to exaggerate your problems to get a place on the priority list.” [P 1]

From the patients' perspective, there was also a concern about the use and storage of the information they provided to the AI application, as well as whether the information could have any future impact on them. This perceived concern influenced how comfortable they felt when providing information.

### 3.2 Alignment with clinical expertise

Trust in the use of the AI-based triage application was related to the participants' experiences of the AI application's alignment with their values and understanding of clinical expertise, especially in relation to standardized reasoning and experience-based knowledge.

#### 3.2.1 Standardized reasoning

Trust in the AI application was experienced by both healthcare professionals and patients as influenced by its standardized reasoning and the simplification of the patients' conditions. While this approach can streamline certain processes, it was seen as not always applicable in primary care, where patients often present complex and multifaceted conditions. Both healthcare professionals and patients viewed patient holistically beyond the biomedical domain, and they agreed that it is not enough to only rely on the linear and standardized reasoning of AI and emphasized the need for a more thorough exploration of the patient's condition.

“It's not specifically about having diabetes or having a high blood pressure or that you have a specific diagnosis, it's more about having some type of health problem. That's more about the lifestyle one has and the society we live in, and it influences us in different ways.” [HCP 3]

“It's like someone sitting and just looking through a care support, ‘nausea, what causes that? Yes, then it may be because of this.’ And then you miss the context or this little word ‘pregnant’ (…) the context is completely lost when you must answer one question on one line.” [P 8]

Another commonality among healthcare professionals and patients was their experience of the AI application following the medical logic and directing the attention towards a specific diagnosis. Its skewness towards treating medical symptoms was experienced by some participants as increasing the medical prescriptions. One healthcare professional explained that the AI application could influence patients to wrongly believe they had a certain diagnosis, which could later be difficult for healthcare professionals to change and could even result in conflicts. From the patients’ perspective, the AI application's standardized reasoning forced them to answer often irrelevant questions, especially if they had more complex or serious conditions. They shared values such as being able to ask questions, being listened to and taken seriously, and that the dialogue should be adjusted according to their unique needs so they could be guided in their understanding. One patient argued that in comparison to healthcare professionals, the AI application did not explain why certain information was necessary or why it acted in a certain way. This was also experienced by healthcare professionals who highlighted the importance of dialogue, asking relevant questions at the right time, and starting with questions of low priority rather than high.

“But take the issue of suicide. In one way I find it a bit strange, it's a matter of trust. An AI is not interested in whether you want to commit suicide or not, so to speak. I would like to be able to ask that question as a psychologist when appropriate.” [HCP 2]

“At this point, I was in a lot of pain, so it felt like someone spat straight into my face when it started asking ‘can you rate your pain?.’ And I only felt, what's this? (…) I only remember the feeling inside when someone asked me that. You're so trapped in your pain that you can barely think, because that's what happens when you're in so much pain. Patients with pain are not a good group to ask that question.” [P 5]

#### 3.2.2 Experience-based knowledge

Experience-based knowledge was frequently reported by healthcare professionals as influencing trust in the AI application but was not mentioned by patients. Healthcare professionals experienced that the suggested diagnoses from the AI-based triage application were more useful for those with less professional experience. Inexperienced professionals often have more focus on symptoms and rare conditions where the AI application was more useful and could assist in identifying and ruling out potential diagnoses, as well as making patient consultations more focused and efficient, while also serving as a helpful reminder.

The healthcare professionals expressed that extensive experience and confidence in their own competence was necessary in order to prevent blind trust in the AI application. They also explained that there is a risk of losing knowledge when not practicing. One healthcare professional explained that professionals with more experience have a broader frame of reference, allowing them to critically assess the suggested diagnoses. At the same time, they experienced a risk of having too much information, which was also experienced as leading to blind trust in their own previous experience.

“Many patients feel that they aren't taken seriously, that nobody listens to them, ‘why does nobody listen?.’ It was quick, the doctor quickly determined that it was this, but I have the feeling that's not what it is, it's something else that's wrong. And this is because you have received too much information and then you just do as you usually do.” [HCP 11]

Trust in the AI application was also influenced by the constant improvement of its predictions based on its “learning” from feedback provided by healthcare professionals.

I imagine, the more you work with it, and it gets more input how to interpret things, or it does not interpret, it doesn't have a brain, but because of its algorithms, it can learn. [HCP 13]

### 3.3 Supervision of patients’ health and safety

Trust in the AI-based triage application is related to the participants' experiences concerning supervision and clearly defined responsibilities in the use of the AI-based triage application. The participants emphasized the importance of ensuring that these systems operate under structured oversight and clear accountability. The theme includes professionalism as a protection against inaccurate information and guidance in the use of the information.

#### 3.3.1 Professionalism as protection against inaccurate information

Both healthcare professionals and patients emphasized the importance of healthcare professionals' safeguarding roles, ensuring that the AI recommendations are appropriately assessed. Many, particularly patients, experienced that there is no need to question the AI application's output, since it is used within a trusted healthcare system with established routines and guidelines. Participants from both groups experienced that healthcare professionals provide a layer of expertise that AI systems alone cannot provide, reinforcing trust in the overall process by validating or adjusting AI outputs based on their clinical knowledge.

“We still have to do everything correctly since the output from the algorithm often has nothing to do with reality” [HCP 3]

“… I just take for granted that they know what they are doing. It may be naïve to think like that, but if I can't trust them, who can I trust. Otherwise, it gets a bit frightening to go down the rabbit hole and start questioning.” [P 6]

Furthermore, one healthcare professional explained that their responsibilities in accordance with “best practice” includes keeping up with the latest applications and deciding whether the AI application is useful or not.

“I try to keep up pretty much, but then there is quite a lot I don't use, but I still think it's our responsibility within the profession to stay on top (…) what if a new drug comes out, a doctor has to know this.” [HCP 2]

#### 3.3.2 Guidance in the use of the information

Both healthcare professionals and patients experienced the importance of clear guidelines in the use of the AI application. From the healthcare professionals' perspective, the information from the AI application was overwhelming and could therefore result in unclear responsibilities and anxiety, as well as overdiagnosis and increased medical prescriptions. The patients' uncertainty concerned who was the receiver of the information and did they use the information. Hence, there were uncertainties regarding the use of the information among both healthcare professionals and patients.

“We can't have that responsibility, but what if the patient writes in the appointment that ‘I will jump in front of the train tonight’ (…). What if I looked ahead a little and something like that was written, then I might have to act.” [HCP 1]

“…this chatbot is a separate system, but I don't know if it actually is (…) but this was somehow confirmed when I met the doctor who started to ask all the questions all over. Well, the questions I answered were not saved nor forwarded.” [P 7]

Some healthcare professionals experienced the risk that the AI application would become a false sense of security. For example, one healthcare professional stressed the importance of confirming the answers with the patients as they may have answered the questions incorrectly or simply ignored answering them. On the other hand, some healthcare professionals explained that the main reason for reading the output from the AI application was out of respect for the patients. From the patients' perspective, they experienced the risk of expecting healthcare professionals to have received and read all the information from the AI application when they in reality had not.

“quite a few have asked me if I have read what they have written in the chat. It's interesting that they want to know that the time they've spent answering the questions is appreciated.” [HCP 4]

“…it's when I'm not insecure that it could potentially become a problem. Because then I don't say what I wrote, and then neither I nor the doctor will know. So, it's necessary that I'm worried and bring it up, which ultimately may create insecurity that this doctor didn't understand me, or the doctor took a decision, and it was wrong because he or she didn't have the information that I thought he or she had. But then and there my insecurity means that it doesn't become a problem, which may not be good.” [P 10]

## 4 Discussion

Our analysis of the interviews with healthcare professionals and patients generated three types of influences on trust in the use of AI-based triage in primary care: provision of accurate patient information, alignment with clinical expertise, and supervision of the patients’ health and safety. Both healthcare professionals and patients reported the importance of communication between patients, healthcare professionals and AI. Trust has traditionally been based on the interaction between healthcare professionals and patients (64). Yet, the use of AI-based triage in primary care forms a trust relationship consisting of healthcare professionals, patients, and the AI-based triage system. This new relationship transforms the flow of information and thereby also the trust relationship between patient and healthcare professionals.

Trust research in healthcare has a tendency to only consider healthcare professionals as trustees in the provision of care, and patients are often overlooked in empirical studies (54, 65, 66). However, the patients' trustworthiness becomes apparent in the use of AI-based triage in primary care. The results showed that trust in the AI application depends on whether the patients are regarded as empowered sources of information. Both healthcare professionals and patients in this study experienced uncertainties whether information could get lost in the use of the AI application due to patients’ lack of competence, but also their willingness to provide information. The patients' empowerment and inner security thus require both education and training, as well as transparency and clear guidelines concerning the care process, use and handling of the patient information.

The results also pointed to the importance of AI's alignment with values of clinical expertise, hence the users' situated knowledge, which draws upon their interactions with the context. The AI application's lack of sensitivity toward the patients' unique needs can restrict its alignment with key values of person-centered care (48, 64). The results suggest the importance of dialogue and aligning the AI application's interactions with the needs and level of knowledge among its users (67). Hence, the importance of considering the values of person-centered care which agrees with previous studies (11, 21). Especially among patients with more complex conditions who require dialogue and understanding, and not only medical competence based on efficiency and accuracy. Previous research (68) shows that the use of AI is influenced by the way the information is communicated and needs to be adapted to the user. The AI application's anthropomorphic characteristics such as human appearance, self-consciousness, and emotions could therefore enhance trust by generating a sense of social presence (40).

Experiential knowledge is sometimes considered a resource for coping with uncertainty in situations of high complexity (69). In this study, extensive professional experience was reported as necessary to be able to critically evaluate the diagnostic suggestions generated by the AI application. However, such experience may have both facilitating and constraining effects on trust. On one hand, it can strengthen professionals' confidence in assessing AI-generated outputs, reinforcing their ability to make informed decisions in familiar clinical contexts. On the other hand, a strong reliance on established routines and past practices may foster skepticism toward new technologies, potentially reducing openness to the innovative solutions offered by AI. This dynamic may explain the participants' concern regarding the application as a false sense of security.

The healthcare professionals thus perceived that long professional experience together with large amount of information and time pressure encourages “blind trust” or what Lebovitz et al. (70) call unengaged “augmentation”. This is in line with trust as a reduction of complexity (71), which can be compared to previous research (24), showing that the use of AI-based triage is influenced by confirmation bias. According to the HCT model (36), emotional responses play a bigger role when there is limited knowledge to make decisions based on cognition. Furthermore, people are often more skeptical of AI when pre-held beliefs are challenged (11, 15). The output from the AI application can thus both facilitate and challenge the healthcare professionals’ decision-making. The differences in the results thus stem from various circumstances and contexts, such as professional experience and the complexity of the patients' health condition. These results may be compared with Calisto et al. (67) who found that AI communication strategies can enhance clinical decision-making and trust in diagnosis if adapted to the clinician's expertise.

In order for AI-based triage to add value to the decision-process, the users must know its boundaries in terms of its capacity and responsibilities related to its use (72). Some healthcare professionals in this study experienced increased uncertainty in the use of the AI application as they experienced a lack of time and resources to address the large amount of information, as well as the risk of overdiagnosis and increased medical prescriptions. Healthcare professionals explained that information only becomes meaningful and useful if there is capacity and resources to address it. Furthermore, the findings in this study suggest that the additional information from the AI application entails new professional responsibilities with uncertainties whether information could get lost in the use of the AI application due to lack of guidelines. Healthcare professionals within a trusted healthcare system with established routines and guidelines function as support and the supervision of patients’ health and safety since they have moral values to decide whether the actions are in accordance with the values of the wider normative system (30).

The findings from this study suggest that trust in AI-based triage is influenced not only by the AI application's performance but also by how users interact with and interpret the flow of information it provides. People's previous experience shape how individuals perceive and process this information, with knowledge serving both as a facilitator and a potential barrier to trust (73). Effective integration of AI-based triage in primary care requires users to possess both competence and confidence in handling and applying the information generated by the AI application. This study deepens our understanding of AI's impact on information-sharing between healthcare professionals and patients, as well as its role in supporting clinical expertise.

### 4.1 Strength and limitations

The study has some strengths and shortcomings that must be considered when interpreting the findings. AI-based triage is viewed as a sociotechnical application and must be understood within the specific context. The study was conducted with the Swedish primary care system, which has unique structural and cultural characteristics. This limits the applicability of findings to other healthcare systems with different regulatory framework and AI adoption levels. Trust in AI-based triage in primary care in Sweden must be understood in the specific context where the interaction between human and AI takes place rather than providing a general overview on trust in AI. The findings reflect the participants' experiences with a single AI application, which may differ from other systems with varying design, functionality or implementation strategies. The study included 14 healthcare professionals and 12 patients, which may not fully represent the diversity of experiences across different healthcare settings. The use of voluntary participation may also introduce selection bias, as individuals with particularly strong opinions or experiences may have been more inclined to participate. Although a larger number of participants with varied backgrounds could have provided a more comprehensive analysis, this study was in line with guidelines on how to conduct a reflexive thematic analysis (58, 59) with both depth and breadth in the data. Participant demographics such as education, age and gender were documented, but more detailed contextual data such as digital literacy or health literacy could have offered deeper insights into how trust in AI is shaped. Reflexivity was a major part of the analysis, which means that we were aware of our previous experiences and their influence on the results. Our multidisciplinary background of competencies, including both clinical and scientific expertise, thus resulted in a profound development of themes.

The study also has considerable strengths. It provides a comprehensive analysis of trust in AI-based triage by considering the perspectives of both healthcare professionals and patients with firsthand experience of AI in clinical practice. This dual perspective allows for a nuanced understanding of how trust is formed and maintained. Furthermore, by incorporating patients' perspectives, the study contributes to addressing the existing knowledge gap concerning patient experiences with AI in healthcare, particularly in the context of applications related to medical history-taking and triage (16). The study benefits from a diverse team of researchers with expertise in nursing, health sciences, sociology, and implementation science. This multidisciplinary perspective strengthens the interpretation and depth of the analysis. The study offers valuable insights into how AI is integrated into real-world healthcare settings, highlighting key challenges and facilitators for trust.

### 4.2 Implications and future directions

This study shows that the use of AI in healthcare raises many complex challenges that are not fully understood. The EU AI Act, the first comprehensive regulation on artificial intelligence (74), emphasizes the need to maintain human control in the use of AI. These results suggest the importance of providing clear roles and responsibilities among the users and specifying what specific competences are needed, as well as tailoring the interaction with AI according to these competences. Hence, the focus needs to be both on competence, as well as transparency and clear guidelines to empower the users and to facilitate trust. Furthermore, in order to ensure safe and effective use of AI, it is also necessary to clarify for what cases AI applications are suitable. These findings can guide the development of more effective AI triage systems and shape implementation strategies that account for the trust dynamics between the technology, patients, and healthcare providers.

Our results pave the way for future research to further explore trust in AI among other AI applications, users, and settings to build trust-strategies and enhance adoption in healthcare. In addition, healthcare professionals in our study had extensive experience within the profession. Future studies could explore trust among healthcare professionals with less experience to reach a deeper understanding of how trust in the use of AI relates to clinical expertise. Comparative studies across various settings and perspectives could inform effective implementation strategies and policy by providing valuable insights into both universal and context-specific influences on trust compared to more general influences.

## 5 Conclusions

Despite the importance of trust in AI for its integration and advancement into healthcare, there are still limited studies exploring the actual use of AI in healthcare. This study provides an in-depth analysis of influences on trust in the use of AI-based triage in primary care, as viewed by healthcare professionals and patients. Overall, the findings based on interviews with healthcare professionals and patients in primary care emphasize the significance of user competence, constructive dialogue between professionals and patients, and the establishment of a trustworthy institutional setting. The results suggest that the user's specific needs must be considered in order to comply with the values of person-centered care. Furthermore, the users need clear instructions and guidelines to address uncertainties and challenges in the new flow of information which arises through the use of AI. New roles and responsibilities that arise must also be carefully considered. Future research should further explore trust in other AI applications and healthcare settings to reach a deeper understanding of the critical uncertainties and challenges that arise in the collaboration with AI to inform policies for safe interaction and use of AI.

## Data Availability

The datasets presented in this article are not readily available because of ethical considerations. Requests to access the datasets should be directed to emilie.steerling@hh.se.
